# Developmental Signals in the 21st Century; New Tools and Advances in Plant Signaling

**DOI:** 10.3390/genes12111708

**Published:** 2021-10-27

**Authors:** Ignacio Ezquer, Paola Vittorioso, Stefan de Folter

**Affiliations:** 1 Department of Biosciences, University of Milan, 20133 Milan, Italy; 2 Department of Biology and Biotechnology C. Darwin, Sapienza Università di Roma, P.le Aldo Moro 5, 00185 Rome, Italy; paola.vittorioso@uniroma1.it; 3 UGA-LANGEBIO, Centro de Investigación y de Estudios Avanzados del Instituto Politécnico Nacional (CINVESTAV-IPN), Irapuato CP 36824, Mexico; stefan.defolter@cinvestav.mx

This special issue includes different research papers and reviews that studied the role of signaling cascades controlling both plant developmental processes and plant response mechanisms to biotic and abiotic stresses. It is not possible to include all relevant areas in a single issue, but this special issue includes excellent research and thought-provoking review articles covering aspects of signal transduction impacting development: transcriptional regulation, hormonal control, cell wall homeostasis, ROS/Ca^2+^ signaling, metabolic programing, etc. Interestingly, authors highlighted important challenges and provided a sharp focus on technical aspects of current research. Those works, which include multiple plant systems (Arabidopsis, grapevine, ferns), focused on many and different developmental processes, such as flower morphogenesis, gametogenesis, plastid differentiation, root development, and seed germination, which overall could orient the direction of future research and efforts in this exciting field.

An emerging new field in plant development concerns the study of signaling by cell wall (CW) modifications, which are responsible for many aspects of plant life. Plant development is extensively studied at many levels. At the cellular level, an important aspect of plant development is the CW. Developmental processes can influence CWs, but also CWs can affect plant development. In this issue, two reviews [1,2] and a research paper [3] studied these aspects. In the work of Cruz-Valderrama et al., 2021, authors reviewed what is known about the importance of the CW during flower development [2]. A focus was placed on the main components of the primary cell wall—cellulose, hemicellulose, and pectins. While cell wall integrity (CWI) signaling has been genetically dissected in other biological models, such as yeast, in plants the heterogeneous and complex structural patterns of CWs made particularly challenging the investigation of CWI signaling in multiple developmental processes. The work by Seifert [1], presented in this special issue, develops for the first time a dedicated and extremely comprehensive review of CWI. This review also provides a specific focus on the FLA4-FEI pathway in order to give an updated state of the art view into the current models of the FLA4-FEI role for CWI control. The work discusses the role of FLA4-FEI in multiple CW aspects, such as the control of pectin-cellulose, and the impact on developmental processes, for instance, on seed formation as well as in signaling pathways, such as the response to cell wall damage (CWD). Interestingly, the work also deals with how CWI is tightly linked to hormone signaling and suggests a role for FLA4-FEI in triggering a set of stress responses, including alterations of auxin, ethylene, jasmonic acid, and salicylic acid levels. The review also provides an interesting tool for further research on the topic, providing readers an excellent overview of the molecular components of the FLA4-FEI pathway [1]. A fruitful aspect discussed in the work involves the issue of the impact of the FLA4-FEI pathway on agronomic traits, such as improving plant resistance to several abiotic and biotic stresses in multiple crops. In line with this, as reported in this issue by Cuadrado-Pedetti et al. [3], drought conditions cause stress to the plant, and the plant will try to adapt to these conditions. A large number of genes have been identified that allow plants to face these conditions; several of these genes have a function in root development. Among these, there is the Arabidopsis *TETRATRICOPEPTIDE THIOREDOXIN-LIKE 1* (*TTL1*). The new study reports novel insights into the function of TTL1. Based on mutant analyses, TTL1 is involved in determining the stiffness of the cell wall of the cells from the root elongation zone. The authors concluded that TTL1 is involved in anisotropic root growth during osmotic stress adaptation [3].

Transcription factors (TFs) are crucial regulators of plant development. This issue includes several articles that discuss the role of TFs in development in distinct plant systems such as Arabidopsis and grapevine [4,5,6]. TFs regulate hundreds to thousands of genes, thereby guiding plant development and signaling processes. This control also has a big impact on the metabolome of the plant. The work reported by Lazcano-Ramírez et al. describes the effect of the induction of the Arabidopsis AP2 TF BOLITA (BOL), which can reprogram plant cell identity, acting on the metabolome and transcriptome [5]. The work identified various enriched metabolic pathways related to the biosynthesis of flavonoids and glucosinolates following BOL induction [5]. Seed development is controlled by a complex, intricate, but also coordinated molecular network controlled by multiple TFs, hormones, and other signal molecules, leading to the formation of its different genetically distinct components, such as the maternal tissues (seed coat) or the “zygotic” tissues (embryo and endosperm). In the research by Paolo et al., multiple approaches are presented in order to understand the interactions between three key TFs that affect seed size, shape, and metabolic processes in the seed coat, using *Arabidopsis thaliana* as a model system [6]. Authors studied in detail the role of STK, GOA, and ARF2, which are important TFs involved in seed formation. Genetic and developmental characterization of the single and multiple mutants revealed that STK, GOA, and ARF2 interact genetically in seed development. The authors demonstrated complex interactions between these regulators and describe some synergistic functions for these factors in the control of processes such as cell expansion and the production of proanthocyanidins. The authors also proved an antagonist function in the control of cell proliferation and release of mucilage. The manuscript presented by Arrey et al. [4] is an excellent research work performed on the reproductive development of grapevine. The work presents the role of the C2H2 zinc-finger proteins (ZFPs), which are involved in pollen development in plants. The work covers multiple agronomic aspects of interest, since, in grapevine, the alteration of pollen development can lead to the phenomenon of parthenocarpy, which occurs in some varieties of this cultivar. The work is novel in the field since the regulatory mechanisms controlling pollen formation in grapevine have been poorly studied so far. To study the role of ZFPs in grapevine pollen development, the authors performed a genome-wide identification and functional characterization of genes encoding ZFPs (*VviZFP* family). They identified 98 potential *VviZFP* genes, and tissue-specific expression pattern analysis allowed them to identify a group of interesting ZFP candidates linked to pollen development. The authors performed elegant expression analysis and described the spatio-temporal expression pattern of two ZFPs during anther and mature pollen grain maturation. These results constitute an important basis for further agronomic studies that can exploit the role of *VviZFP*s in grapevine reproductive development [4].

This issue also includes an interesting review on reproductive development [7]. The authors focused on the seed-to-seedling transition, which is a key step in plant life, strictly controlled by both endogenous and environmental cues. Indeed, seedling development should occur when autotrophic growth is likely. The molecular networks that control these developmental steps have been deeply investigated. The review by Longo et al., 2021 [7], focuses on new molecular elements in the transcriptional, translational, and epigenetic regulatory mechanisms, pointing out how these networks integrate the response to environmental and hormonal signals [7]. Attempting to provide a more complex view of the different developmental processes affecting plants, this issue includes an excellent research paper focused on the process of differentiation of functional chloroplasts [8]. This complex process, as reported in this work, is delimited by the optimal balance between the rate of plastid protein synthesis and the activity of the plastid protein quality control machinery. Development and function of chloroplasts are crucial for plant growth; abnormal or dysfunctional chloroplasts undergo degradation through a tightly controlled process which involves protein ubiquitination. By means of a genetic approach, aimed at suppressing the leaf variegated phenotype of the *gun1-102 ftsh5-3* double mutant, Jeran et al., 2021 [8], identified the Plant U-Box 4 (PUB4) E3 ubiquitin ligase as a component of the chloroplast quality control machinery. Indeed, *PUB4-7* inactivation, in the *gun1-102 ftsh5-3* background, is able to restore functional chloroplasts, although with an altered phenotype, thus suggesting that the variegated phenotype of *gun1-102 ftsh5-3* is largely dependent on PUB4 activity [8].

Interesting views are also provided in this issue by including developmental studies using other model systems, such as ferns [9]. A crucial question in plant developmental biology relates to the tight control of the balance between cell proliferation and cell expansion to modulate organ growth and shape organ size. The root represents the most suitable organ for evo-devo studies, in particular the root apical meristem, which is a peculiar trait in ferns compared to seed plants. In this issue, Aragón-Raygoza et al. present a study on root development in *Ceratopteris richardii* (Ceratopteris), a subtropical fern which represents a plant model system for developmental biology studies. The results of this work support the hypothesis of a high mitotic rate of the root apical cell, while suggesting the lack of a quiescent center in the stem cell niche of Ceratopteris roots [9].

Reactive oxygen species (ROS) control multiple developmental processes, and in this issue two complementary contributions reviewed these relationships [10,11]. Different enzymes participate in plant development and stress signaling. One group of enzymes is called the plant aldehyde dehydrogenase enzymes (ALDH). Stress to plants causes the formation of ROS, which in turn causes the excessive accumulation of aldehydes in cells. ALDH enzymes metabolize aldehyde molecules. In this issue, Tola et al. reviewed the recently discovered roles of these enzymes during plant development and stress signaling in plants [10]. ROS and Ca^2+^ signaling pathways also determine gametophyte functioning, sexual reproduction, and embryo formation in plants and animals. In this issue, Lodde et al. [11] proposed an integrative and comparative discussion around studies on the role of ROS/Ca^2+^ in both plant and animal developmental biology studies to further elucidate these crucial signaling pathways. The field is well explored in animals, and, in recent years, multiple advances in plant science have been made concerning signal transduction via ROS and Ca^2+^ signaling into developmental processes and in response to biotic and abiotic stresses. The emphasis on the reproductive system provided in the work is interesting, since this had not previously been reviewed. The review described the basis for ROS production, metabolism, and detoxification systems used by plants and animals to control ROS homeostasis below toxicity levels. Interestingly, the authors reviewed recent developments in the use of genetically engineered sensors to monitor concentration fluxes and localization of ROS/Ca^2+^ in vivo [11]. These concepts and ideas have great potential and interest for researchers working in the field and offer valuable up-to-date tools to monitor ROS signaling in vivo, both in plant and animal systems. The review also focused on the reproductive systems and ROS/Ca^2+^ signaling pathways involved in animal embryo and seed development and discussed “omics” data, providing a list of potential targets affecting ROS in reproductive processes in plant development [11].

We would like to express our great appreciation for the efforts of the 64 authors from 20 institutions (in nine different countries) that have participated in this special issue, even more so considering the difficult circumstances that have accompanied the pandemic (this special issue started in February 2020) and the limitations researchers have suffered to keep science active in the laboratories. We also thank enormously the work of the reviewers involved in the revision of the 11 articles that constitute this issue.

In summary, the current Special Issue “New Insights into Plant Development and Signal Transduction” provides a state-of-the-art perspective of experts in the field and also provides suggestions for potential industrial applications and challenges for plant biologists.
